# Supplementary material for: Comparison of CLEIA and ELISA for SARS-CoV-2 Virus Antibodies after First and Second Dose Vaccinations with the BNT162b2 mRNA Vaccine
Source: Vaccines (Basel). 2022 Mar 22;10(4):487. doi: 10.3390/vaccines10040487 (PMC9033149; doi:10.3390/vaccines10040487)
Supplement: Supplementary file 1 [file vaccines-10-00487-s001.zip › vaccines-1627843-supplementary.pdf]

Comparison of CLEIA and ELISA for SARS-CoV-2 virus anti-bodies after first and second dose vaccinations with the BNT162b2 mRNA Vaccine

Mohammad Said Ashenagar[1]*, Akiko Matsumoto[1]*, Hironori Sakai[2], Mikiko Tokiya[1], Megumi Hara[3], and Yoshio Hirota[4]

Supplementary Files

Table S1. Reproducibility of antibody test results using ELISA based on the optical density (O.D.) at 450 nm.

	S1 IgM		S1 IgG		NP IgG	
Level	L	H	L	H	L	H
Absorbance (O.D. 450 nm)						
Mean	0.05	1.25	0.05	1.06	0.06	1.29
SD	0.00	0.16	0.00	0.10	0.00	0.13
CV (%)	6.5	13.0	6.2	9.5	5.3	9.9

Control samples were repeatedly measured ten times by each assay. SD, standard deviation. CV, coefficient of variation. S1, anti-spike protein S1 subunit antibody; NP, anti-nucleocapsid protein antibody. L, low concentration control; H, high concentration control.

Table S2. Number of blood samples taken from the participants.

Week after first dose	0	1	2	3	4	5	6	7	11	15	SUM
Healthcare workers	20	20	19	19	18	19	19	19	19	19	191
Hospital staff	–	–	–	–	–	–	–	59	–	–	59
Students	42	–	–	42	–	–	–	42	41	41	208
SUM	62	20	19	61	18	19	19	120	60	60	458
